# Understanding the role of steroids in typical and atypical brain development: Advantages of using a “brain in a dish” approach

**DOI:** 10.1111/jne.12547

**Published:** 2018-02-18

**Authors:** D. Adhya, E. Annuario, M. A. Lancaster, J. Price, S. Baron‐Cohen, D. P. Srivastava

**Affiliations:** ^1^ Department of Psychiatry Autism Research Centre University of Cambridge Cambridge UK; ^2^ Department of Basic and Clinical Neuroscience Maurice Wohl Clinical Neuroscience Institute Institute of Psychiatry, Psychology and Neuroscience King's College London London UK; ^3^ MRC Laboratory of Molecular Biology Cambridge UK; ^4^ MRC Centre for Neurodevelopmental Disorders King's College London London UK; ^5^ National Institute for Biological Standards and Control South Mimms UK

**Keywords:** 17β‐oestradiol, androgens, autism spectrum conditions, neurodegenerative disease, neurodevelopmental disorders, oestrogens, psychiatric, schizophrenia, stem cells, synapse, testosterone

## Abstract

Steroids have an important role in growth, development, sexual differentiation and reproduction. All four classes of steroids, androgens, oestrogens, progestogens and glucocorticoids, have varying effects on the brain. Androgens and oestrogens are involved in the sexual differentiation of the brain, and also influence cognition. Progestogens such as progesterone and its metabolites have been shown to be involved in neuroprotection, although their protective effects are timing‐dependent. Glucocorticoids are linked with stress and memory performance, also in a dose‐ and time‐dependent manner. Importantly, dysfunction in steroid function has been implicated in the pathogenesis of disease. Moreover, regulating steroid‐signalling has been suggested as potential therapeutic avenue for the treatment of a number of neurodevelopmental, psychiatric and neurodegenerative disorders. Therefore, clarifying the role of steroids in typical and atypical brain function is essential for understanding typical brain functions, as well as determining their potential use for pharmacological intervention in the atypical brain. However, the majority of studies have thus far have been conducted using animal models, with limited work using native human tissue or cells. Here, we review the effect of steroids in the typical and atypical brain, focusing on the cellular, molecular functions of these molecules determined from animal models, and the therapeutic potential as highlighted by human studies. We further discuss the promise of human‐induced pluripotent stem cells, including advantages of using three‐dimensional neuronal cultures (organoids) in high‐throughput screens, in accelerating our understanding of the role of steroids in the typical brain, and also with respect to their therapeutic value in the understanding and treatment of the atypical brain.

## 1 INTRODUCTION

Steroid hormones are cyclical chemical compounds made up of rings of carbon atoms that play an essential role in a wide range of physiological functions, including growth, development, energy metabolism, homeostasis and reproduction. Within the brain, these molecules exert profound effects on brain development, sexual differentiation, reproductive behaviour and cognition, including learning and memory.^1^, ^2^, ^3^, ^4^, ^5^ Traditionally, steroids have been thought to exert their actions by binding to classic nuclear receptors located within cytoplasm, which then translocate to the nucleus and regulate gene transcription over a period of hours to days. The crucial structural and functional effects that steroid hormones have on the brain have made them prime targets for studying mechanisms associated with gene regulation. For example, a high dose of steroid hormones where the levels are assumed to be low, and an absence where there is supposed to be high levels of a hormone, have transformative effects on the physiology of the brain.^2^, ^6^ However, it is now becoming increasingly apparent that many of the actions that steroids exert occur within a time frame too rapid to be accounted for by this mode of action. Indeed, many steroid nuclear receptors and several novel steroid receptors have been found at the plasma membrane, where they are considered to engage with signalling proteins.^3^, ^4^, ^7^, ^8^ The increasing appreciation of the contribution that such ‘membrane‐initiated’ actions have on how steroids impact brain function has also led to suggestions that ‘neurosteroids’ may need to be regarded more as neuromodulators, or transmitters in addition to hormones.^9^, ^10^ Interestingly, dysregulation of all four steroids classes have been implicated in a range of disorders, including stress, autism‐spectrum disorders, schizophrenia and Alzheimer's disease, to name but a few. Moreover, there is continued interest in the use of steroids as potential therapeutic agents.^1^, ^3^, ^11^, ^12^, ^13^, ^14^ In this review, we use ‘atypical brain’ to describe abnormal brain development or function that contributes to a specific disorder.

To date, much of our understanding of the molecular and cellular mechanisms by which steroids exert their effects within the brain have originated from in vitro and in vivo animal based models. Such studies have provided a fundamental understanding of the role of steroids in neural tissue; however, the effects of these molecules and the underlying molecular mechanisms in human neural tissue are not well understood. Ethical considerations make working with human tissue a challenge, if not impossible in certain cases. Although several human neuroblastoma and immortalised neural cell lines exist, it could be argued that these cellular systems do not faithfully recapitulate a human neuronal cellular environment of specific neuronal linages or inherent differences that would be expected between individuals. Thus, these cellular systems are not fully suited for detailed cellular and molecular studies investigating the mechanisms underlying the effects of steroids in neural cells under typical or atypical states. Recent advances in stem cell biology have now provided us with the ability to generate native human neurons in which to study basic and disease mechanisms.^15^, ^16^, ^17^, ^18^ This has led to the ability to reprogram patient somatic cells into human‐induced pluripotent stem cells (h‐iPSCs) and the subsequent differentiation of these cells into neural cells of specific lineages.^16^, ^17^ Importantly, these cells encapsulate and recapitulate the genetic landscape and cellular abnormalities associated with complex disease.^19^, ^20^ Critically, this approach provides a potentially limitless source of live human cells for understanding basic neurobiology and disease pathophysiology, as well as for modelling the actions of potential drug targets.^16^, ^17^, ^18^, ^21^ This review aims to cover the effects of gonadal and adrenal steroids on the brain, including long‐term effects on sexual differentiation and acute effects on cognition and memory. We aim to do this by giving an overview of the different physiological effects brought about by steroid hormones and then classifying the known nuclear and cytoplasmic mechanisms responsible for those effects. These topics have been greatly investigated and therefore we can only briefly highlight a small selection of studies. In addition, we discuss the effect of dysregulated steroid hormone exposure and its implications in atypical brain states, before going on to explore the possible use of steroid hormones as therapeutics. Finally, we discuss how the use of human iPSCs now offers a novel cellular system in which to better understand the role of steroids in human neurodevelopment, their contribution to disease and therapeutic potential.

## 2 STEROID HORMONES IN THE TYPICAL AND ATYPICAL BRAIN

Although sexual dimorphism in an animal is determined by biological sex, sexually dimorphic behaviour is not a binary phenomenon. It is achieved through a complex cascade of cellular and molecular changes induced by changing levels of steroid hormones in the circulation. Steroid hormones act through nuclear receptor‐mediated mechanisms that cause permanent sexual dimorphism of the brain. However, recent studies have shown that, under certain conditions, steroid hormones also interact with cell surface receptors to cause fast‐acting, acute alterations in brain function. Here, we will discuss the well‐characterised effects of steroid hormones on sexual dimorphism of the brain and the underlying molecular mechanisms. We also will discuss the more acute effects of steroids on behaviour, cognition and memory that act independently of sexually dimorphic mechanisms, and may play major roles in the typical and atypical brain.

### 2.1 Sexually dimorphic brain and behaviour mediated by testosterone‐ and oestrogen‐induced nuclear receptor regulation

Transformative effects of steroids on sexual dimorphism of the brain first starts taking shape in humans during foetal development when there is a surge of testosterone in the male foetus during 8 to 24 weeks of gestation,^22^, ^23^ and this hormone surge is assumed to have long lasting structural and functional effects on the brain. The presence of fetal testosterone (fT) during gestation has been associated with greater grey matter volume in right temporoparietal junction/posterior superior temporal sulcus (RTPJ/pSTS), whereas an absence of fT has been associated with greater volume in planum temporale/planum operculum (PT/PO) and posterior lateral orbitofrontal cortex (plOFC).^23^ Testosterone exposure during the prenatal period also affects the adult sexually dimorphic nucleus of the preoptic area (SDN‐POA), a substructure of the medial preoptic area of the hypothalamus (MPOA) in humans, which is 2.2 times larger in men as in women.^24^ In rodent studies, the spinal cord nucleus (SNB; spinal nucleus of the bulbocavernosus) and the MPOA and especially its substructure, SDN‐POA are also larger on average in males as in females,^25^, ^26^ whereas, on the other hand, the anteroventral periventricular nucleus (AVPV) is larger in females as in males.^2^

There is a hypothesised role of fT in atypical neurodevelopment through associative studies from our group which show significant association of elevated fT levels with autism when compared to typical individuals, and fT levels were positively associated with higher scores on the Childhood Autism Spectrum Test (CAST) and the Child Autism Spectrum Quotient (AQ‐Child).^27^ The brain region RTPJ, which has been shown to have a greater volume with increased fT, is also known to be associated with autism.^28^, ^29^ Additionally, elevated testosterone levels have been positively associated with occurrence of obsessive compulsive disorder, and Tourette syndrome,^30^ whereas elevated levels of gestational oestrogens have been suggested to increase predisposition to schizophrenia.^31^

**Figure 1:**
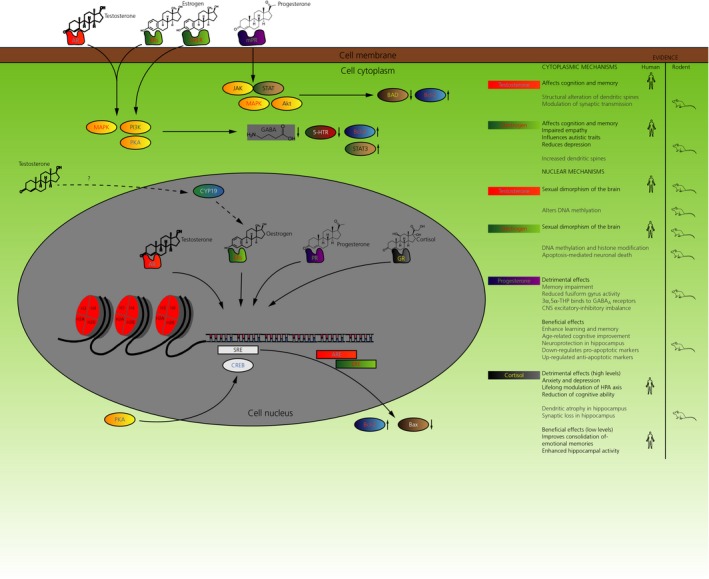
Nuclear and cytoplasmic effects of steroid hormones. Schematic diagram describing the mechanism of actions for all four major classes of steroids. Actions are classified into ‘nuclear’ and ‘cytoplasmic’ (mediated by membrane‐bound receptors) and further described whether such actions are considered to be beneficial or detrimental, if the steroid function is dysregulated. The nuclear mechanism of steroid mediated signalling involves binding of steroid receptors with their corresponding steroid receptor element (SRE). Binding to androgen and oestrogen receptors elements (ARE and ERE) have been associated with regulation of apoptotic molecules, Bax and Bcl2. BAD, Bcl2‐associated death promoter; CREB, cAMP response element‐binding protein; GPER, G‐protein coupled oestrogen receptor; GR, glucocorticoid receptor; JAK, Janus kinase; MAPK, mitogen‐activated protein kinase; MPR, mannose‐6‐phosphate receptor; PI3K, phosphoinositide 3‐kinase; PKA, protein kinase A; STAT, signal transducer and activator of transcription

The mechanism of steroid‐induced behavioural outcomes is not well understood, and why there is increased steroid hormone levels in individuals with neuropsychiatric conditions even less so. Research using animal models have identified two major pathways for long‐term sex steroid effects mediated by steroid receptors: apoptosis and epigenetic modulation. Testosterone and oestrogens also induce acute changes to neuron and synapse structure through a protein kinase‐mediated mechanism, as discussed below. Sex‐specific behaviour is mediated through nuclear receptor mechanisms (Figure 1) and, according to the classical model, testosterone and androgen receptor (AR) binding has been shown to be responsible for sex‐specific behavioural effects in males.^32^, ^33^ There is additionally a parallel mode of sexual differentiation involving oestrogens, and a loss of both oestrogen receptors (ERα and ERβ) has been shown to cause atypical male‐specific behaviour in rats^34^ and, in males, ERα is thought to augment male sexual behaviour, whereas ERβ is thought to supress female sexual behaviour.^35^, ^36^

#### 2.1.1 Programmed neuronal death

The hormonal control of cell death is the most well‐established mechanism associated with sexual dimorphism of the brain.^37^ Studies in rodent models have revealed sex‐specific activation of apoptosis in particular subsets of neurons.^34^ Reduced neuronal death has been shown to be responsible for larger SNB and MPOA in males.^25^, ^26^, ^38^, ^39^ Neuronal death in these brain regions in females is ameliorated by treatment with oestrogens or testosterone,^25^, ^38^ resulting in a permanent reversal and masculinisation irrespective of biological sex. It is thought that aromatase (CYP19), which is responsible for the synthesis of oestrogens from testosterone and is actively expressed in brain regions involved in sexual differentiation,^40^, ^41^ plays an important role in masculinisation, and the use of aromatase inhibitors has revealed reduced male‐typical sexual behaviour in rats. Interestingly, it has been shown that apoptosis‐mediated cell death is not responsible for sexual differences in the ovine SDN (a substructure of the MPOA‐equivalent brain region in sheep).^42^ The sexually dimorphic brain region AVPV is larger in females as a result of the unusual nature of oestrogen activity, which, in the male AVPV, triggers apoptosis of neurons via the activity of both the ERα and ERβ.^2^, ^43^, ^44^

Mechanisms causing testosterone and oestrogen‐mediated sexually dimorphic neuronal death include the modulation of Bcl‐2 (anti‐apoptotic factor) and Bax (pro‐apoptotic factor) expression.^45^, ^46^ Oestrogens increase Bcl‐2 levels and decrease Bax levels in the pre‐optic area of rats,^47^ and testosterone is considered to have the same effect in an indirect fashion through aromatisation to oestrogens.

#### 2.1.2 Epigenetic mechanisms

A second major cellular mechanism of steroid signalling involves epigenetic modulation to bring about long‐term organisational effects. Testosterone and oestrogen signalling through AR and ERs interact with histone‐modifying factors such as histone acetyl transferases to bring about profound gene regulatory effects.^6^, ^48^, ^49^ ERα demonstrates high promoter methylation in both sexes corresponding to reduced ERα gene expression in the cortex,^50^, ^51^ and a study using rats has shown that greater ERα promoter methylation is associated with higher neonatal maternal grooming and early oestradiol exposure.^52^ Another study in mice showed that testosterone administration in neonatal females altered brain DNA methylation 20‐fold in adulthood to shift towards a male methylation pattern,^53^ whereas short‐term effects of testosterone were relatively modest. This late‐emerging effect provides insight into the vital role of steroids in long‐term sexually dimorphic molecular changes in the brain, and also shows the importance of the effect of steroids on DNA methylation in bringing about brain sex‐specific organisation.

### 2.2 Testosterone and oestrogens modulate immediate effects on cognition and memory by protein kinase pathways

#### 2.2.1 Behavioural effects

Testosterone and oestrogens are known to have long‐term effects on cortical organisation through mechanisms mediated by nuclear receptors. Current research has also uncovered the cytoplasmic mechanisms of action and localisation of AR and ERs outside the cell nucleus, in membranes, dendritic spines and presynaptic terminals of neurons^4^, ^54^ (Figure 1), showing more immediate kinase‐mediated effects^54^ on behaviour and cognition that are independent of sexual programming during development. Immediate effects of testosterone and oestrogens have been reported in human studies, such as in one study from our group where a single sublingual dose of testosterone administered to young women significantly impaired empathy and social cognition,^55^ although the mechanism of action is not known. Another study reported elevated testosterone levels associated with higher emotional arousal and amygdala activation in males.^56^ In animal model studies, testosterone and oestrogen administration to ovariectomised adult female rats appear to alter sexually dimorphic behaviour in the radial maze and Morris water maze tasks,^57^, ^58^, ^59^, ^60^ whereas oestrogen administration specifically appears to cause acute enhancement of memory consolidation with improvements in spatial and nonspatial memory tasks.^61^ As a result of technical limitations associated with working with human samples, cellular/molecular mechanisms in humans are poorly understood, although some steroid‐mediated mechanisms involving protein kinases have been identified in animal studies, as described below.

#### 2.2.2 Cellular/molecular mechanisms

General mechanisms responsible for testosterone and oestrogen‐mediated learning and memory alterations involve structural and functional modulation of dendrites and dendritic spines. Animal studies have reported that the formation of new memories is associated with oestrogens, which increases dendritic spine density in the CA1 region of the hippocampus, at the same time as causing structural changes to existing spines.^62^ Dendritic spine density in the hippocampus CA1 region has also been shown to increase upon administration of testosterone, and this process is oestrogen‐independent.^63^, ^64^ Previous studies from our group have also established a role for oestrogens in the structural modulation of dendrites in human neurons,^18^ and oestrogens have been shown not only to regulate synaptic transmission, but also to increase physical connections between neurons.^65^ Dendritic spine density is regulated by the interaction of steroid hormones with the extracellular signal regulated kinase‐mitogen activated protein (ERK‐MAP) kinase pathway, and studies in mice have shown that inhibiting phosphorylation of ERK‐MAP kinases completely prevents oestrogen‐associated dendritic spine formation.^5^, ^65^ The effect of testosterone on spine density is mediated via the same mechanism, after conversion of testosterone into oestradiol intracellularly.^66^ In addition to stimulating the formation of nascent excitatory synapses,^4^, ^65^ oestrogens also suppress signalling at inhibitory synapses.^67^, ^68^ This may underlie oestrogen's ability to influence neuronal excitability and seizure disorders.^69^ Oestrogens are also known to induce phosphoinositide kinase and protein kinase A activity through the G‐protein coupled oestrogen receptor (GPER),^4^, ^8^ Activation of GPER1 in developing neurons results in the triggering of neurite outgrowth via a cAMP response element‐binding protein‐mediated transcriptional mechanism.^67^, ^68^ Interestingly, activation of GPER may also be involved in the prevention of tau protein hyperphosphorylation in Alzheimer’s disease,^70^, ^71^ and protect against glutamate excitotoxicity and inhibition of GABA neurotransmission.^72^, ^73^, ^74^ Moreover, signalling via GPER can also result in the rapid desensitisation of serotonin receptors to accelerate the selective serotonin reuptake inhibitor effect^75^, ^76^ and an increased signal transducer and activator of transcription activation associated with the enhanced satiety response.^77^, ^78^ Taken together, the rapid protein kinase‐mediated synaptic effects of testosterone and oestrogens on cognition demonstrate a wider effect of steroid signalling in the brain not related to developmental programming or sexual differentiation.

### 2.3 Additional transformative effect of progesterone and glucocorticoids through nuclear receptor‐mediated mechanisms

#### 2.3.1 Progesterone

##### Neurodeteriorative effects

Progesterone, similar to oestrogens, has neuroprotective effects, and impacts cognitive function (Figure 1).^79^ In some cases, progesterone has been shown to impair cognition and memory and, in other cases, it has been shown to demonstrate an enhancement.^80^ The disruptive effect on memory and cognition is dependent on the timing of progesterone administration. For example, one study reported an improvement in working and spatial memory after ovariectomy in aged female rats, which was reversed on progesterone administration, demonstrating the detrimental effects of ovarian progesterone on the female brain.^81^ Another study using human participants reported memory impairment and reduced response to faces in amygdala and fusiform gyrus after a single administration of progesterone.^82^ The disruptive effect of progesterone is considered to occur through its metabolite allopregnanolone, which binds to GABA_A_ receptors to produce an inhibitory effect and alter the excitatory‐inhibitory balance in the central nervous system (CNS).^83^ The disruptive nature of allopregnanolone‐GABA_A_ receptor interaction also has therapeutic effects, as discussed later below.

##### Neuroenhancing effects

In contrast with the detrimental effects of progesterone described above, many studies have shown memory enhancement following progesterone administration. One study reported prevention of age‐related memory loss in aged overiectomised rats following chronic administration of progesterone along with oestrogens.^84^ In certain cases, the progesterone metabolite allopregnanolone is involved in the recovery of age‐related cognitive deficits.^85^ Progesterone was also shown to enhance learning and memory, and to recover age‐related cognitive decline in aged wild‐type as well as progesterone receptor knockout mice.^86^ A possible cellular mechanism for the role of progesterone as a memory enhancer is its ability to provide neuroprotection against oxidative stress in the hippocampus,^87^ by down‐regulating pro‐apoptotic markers and up‐regulating anti‐apoptotic markers.^88^ Some studies suggest that progesterone‐mediated neuroprotection might be result of rapid membrane‐initiated kinase activity, which down‐regulates pro‐apoptotic factors such as Bcl2‐associated death promoter and up‐regulates anti‐apoptotic factor Bcl2, and this pathway is triggered via a membrane progesterone receptor.^89^, ^90^, ^91^ Although the effect of progesterone on the brain can be two‐pronged, depending on the timing and dose of the hormone, in some conditions, controlled doses of progesterone has been beneficial in ameliorating neurological symptoms, as also discussed later below.

#### 2.3.2 Glucocorticoids

##### Neurodeteriorative effects

Glucocorticoids (cortisol in humans and corticosterone in nonhuman mammals), similar to other steroid hormones, have an important function in the developing foetus, and are required for the normal growth and maturation of the cerebral cortex, the hippocampus and pituitary gland^92^ (Figure 1). Studies in animal models have identified a high level of glucocorticoid‐mediated plasticity in the hippocampus including neurogenesis in dentate gyrus^93^ and structural modulation of dendrites and synapses in the Ammon's horn.^94^ However, similar to progesterone, the glucocorticoid surge must occur at a precise developmental window and at a precise concentration to maintain normal developmental trajectories.^95^, ^96^ Studies have shown that glucocorticoid administration during late gestation can affect structural development of the foetal brain,^97^ including a reduced neuron density in the hippocampus.^98^ Antenatal glucorticoid administration causes thinning of the anterior cingulate gyrus and exacerbates associated affective disorders such as anxiety and depression. Anomalous glucocorticoid exposure is also linked with an increase in the risk of cerebral palsy in preterm infants.^99^ Some of the neurological effects of early glucocorticoid exposure also appear to be transmitted across multiple generations.^100^ In humans, persistent lifelong modulation of the hypothalamic‐pituitary‐adrenal (HPA) axis can cause long‐term fluctuations of blood glucocorticoid level, which can have adverse effects on psychiatric health.^101^ Glucocorticoid‐induced stress can cause reduction in cognitive ability, including deteriorating working and nonverbal memory, mental flexibility and information processing.^102^ These symptoms have been associated with dendritic atrophy and synaptic loss in hippocampal neurons.^103^ Glucocorticoids mediate cellular/molecular effects on the synapse and dendrites through both the glucocorticoid receptor (GR) and mineralocorticoid receptor.^104^ The promoter region of GR is highly sensitive to stress and glucocorticoid administration, and one study has reported increased histone acetylation and DNA demethylation associated with the GR promoter region when stress is low,^105^ increasing GR expression, which is associated with an increased glucocorticoid‐induced negative feedback loop and decreased HPA activity.^106^ Conversely, increased GR promoter DNA methylation has been associated with child abuse victims and people who commit suicide.^107^

##### Neuroenhancing effects

The effect of glucocorticoid‐mediated stress can be beneficial when the individual is exposed to it during a precise window.^108^ For example, glucocorticoid administration just after a learning event facilitates subsequent consolidation of memory, and these effects are particularly strong for emotion‐related memories.^109^, ^110^, ^111^, ^112^ However, presentation of stress after memory retrieval affects reconsolidation, and later recall in the individual can be impaired.^113^, ^114^, ^115^ Additionally, exposure to stress before learning in some cases impairs memory formation^116^, ^117^ and, in other cases, enhances it.^118^, ^119^ Low blood glucocorticoid levels have also been associated with enhanced cerebral infusion, glucose utilisation and hippocampal neuron activity, which are reversed when glucocorticoid levels increase.^120^ This establishes the complicated interaction of glucocorticoid‐mediated stress with cognition and memory, and the importance of administering a low concentrations of glucocorticoid at critical windows to have any beneficial effect on brain function. Further information on this topic is provided by Moisiadis and Matthews^96^ and Sorrells et al.^120^

## 3 STEROIDS AS THERAPEUTIC AGENTS

Of the wide‐ranging functions of steroids, oestrogens and progesterone have consistently been shown to have a neurological ameliorative and enhancing role, and thus have been proposed as potential treatments for certain neuropsychiatric and neurodegenerative conditions. Here, we discuss the ameliorative effects of oestrogens and progesterone, as well as their roles in the treatment of neuropsychiatric and neurodegenerative conditions (Table 1.

**Table 1:** Proposed therapeutic roles of oestrogens and progesterone

Steroid hormone	Organism	Therapeutic role
Oestrogen	Human	Wellbeing and improved cognition in typical and schizophrenic^114^, ^115^
	Rodents	Higher survival of females after traumatic brain injury^116^, ^117^
		Positive neurological outcome in males^118^
		Reduction of apoptosis and enhanced recovery after spinal cord injury^119^
Progesterone/allopregnanolone	Human	Protective effect in Alzheimer's disease^121^
	Rodents	Reduced depression in Parkinson's rat model^122^
		Improved hippocampal structure and function after middle cerebral artery occlusion^123^
		Reduced cell death and DNA fragmentation after traumatic brain injury^129^

### 3.1 Oestrogens

The cognitive enhancing effects of oestrogens, such as 17β‐oestradiol, have highlighted suggestions that oestrogens or oestrogen‐mimicking compounds may be a useful therapeutic agent for neuropsychiatric conditions.^11^, ^14^, ^121^ Studies in humans have shown a higher occurrence of schizophrenia in males, and increased psychosis in females during depletion of oestrogens,^14^, ^121^ and the administration of 17β‐oestradiol or the selective oestrogen receptor modulator, raloxifene, has demonstrated a positive effect on wellbeing and cognition for typical individuals, as well as for individuals with schizophrenia.^122^, ^123^ The mechanisms by which 17β‐oestradiol is beneficial in neuropsychiatric disorders are not well understood, although they are considered to occur partly through modulation of mono‐amine production, including serotonin and dopamine, as well as through modulation of glutamatergic signalling, in addition to anti‐inflammatory effects.^4^, ^121^ In animals, oestrogens also have a mild sexually dimorphic effect on a traumatic brain injury rat model, with some studies showing higher survival in female rats,^124^, ^125^ whereas others report better neurological outcomes in males.^126^ In a rat model of spinal cord injury, oestrogen treatment can significantly reduce apoptosis at the same time as enhancing the recovery of spinal cord functions.^127^

### 3.2 Progesterone

Progesterone and its metabolite allopregnanolone have a neuroprotective function and are able to stimulate neurogenesis and enhance cognition.^128^ They have a more predictable neuroprotective effect, which is not sexually dimorphic, and thus have been more extensively studied than oestrogens as a potential therapeutic agent for neurodegenerative conditions. Progesterone has protective effects on human neurodegenerative conditions such as Alzheimer's disease.^129^ One study showed that the administration of progesterone to a rat model of Parkinson's disease significantly reduced depression‐like symptoms.^130^ Administration of progesterone and allopregnanolone to rats suffering from severe cerebral ischaemia after middle cerebral artery occlusion was found to improve the structure and function of the hippocampus, although the preservation of cognitive functions is significantly better if progesterone was administered before inducing cerebral ischaemia.^131^ It is hypothesised that, even though there is severe neuronal loss in the hippocampus after ischaemia,^132^, ^133^ the neuroprotective action of progesterone is able to enhance structure and plasticity of remaining neurons to condition them to carry out alternative strategies in the hippocampus or connected structures to restore some of the lost cognitive functions.^134^, ^135^ Traumatic injury to the rat frontal cortex has been reported to kill large numbers of neurons, as well as to produce acute inflammation, oedema, astrocyte hypertrophy and blood‐brain barrier compromise,^136^ and progesterone and allopregnanolone have been used to alleviate its effects by reducing cell death and DNA fragmentation, as well as protein expression of pro‐apoptotic markers caspase‐3 and Bax, in addition to astrocyte hypertrophy.^137^ Allopregnanolone has also been successfully used to prevent neuronal death in animal models of stroke and spinal cord injury, and this involves a GABA_A_ receptor‐mediated mechanism.^138^, ^139^, ^140^ The benefits can be usually seen after only a brief period of progesterone administration.

It is thus evident that both oestrogens and progesterone have neuroprotective effects on the brain and therefore may be useful for treating certain symptoms of brain injury, as well as neuropsychiatric and neurodegenerative diseases. The beneficial effects of oestrogens occur via the modulation of neurotransmitters such as serotonin, glutamate and dopamine in the brain, or by directly influencing glutamatergic function. Progesterone and its metabolite allopregnanolone appear to act by reducing apoptosis and a wide range of inflammation responses associated with brain injury, at the same time as structurally transforming hippocampal neurons to assist in the restoration of cognitive functions. However, one major limitation of all these studies is that they have been mostly undertaken in animal models and how these data translate to humans remains unclear. Indeed, there has only been limited success in translating preclinical work into novel therapeutic agents to treat debilitating neurological, neurodevelopmental or neurodegenerative disorders. This lack of conversion is a result of many factors, although these likely include species differences, as well as differences in brain complexity and disease‐specific phenotypes.^141^ One example of this has been the adoption of hormone therapy for neurological conditions in humans. The Women's Health Initiative study reported a decrease in cognitive function and an increased risk of dementia and stroke in women over 65 years of age who received conjugated equine oestrogens plus medroxyprogesterone acetate compared to those who received placebo,^142^ despite an abundance of preclinical studies indicating a neuroprotective role for oestrogens in dementia. Although, the findings of these studies have come under much criticism,^143^, ^144^, ^145^, ^146^ it is nevertheless critical to develop a better understanding of how potential steroid‐based therapeutic agents impact normal physiology in human neurons, as well as in appropriate models of disease to avoid unwanted side‐effects. Although the use of animal models is essential to such studies, the use of additional models that allow investigations in human neurons would be of huge benefit. Recent developments in human stem cell technologies especially in iPSC technology, have enabled researchers to model the human brain in a Petri dish and, as this technology matures, there is the potential that steroid research using human‐derived tissues will accelerate the development of novel hormone therapies for neurological conditions.

## 4 ROLE OF IPSCS IN NEUROSTEROID RESEARCH

### 4.1 From iPSCs to specific neurons

The method of reprogramming adult somatic cells to pluripotent stem cells was first described in 2006 by Takahashi and Yamanaka.^147^ They reported that dermal fibroblasts from adult mice could be reprogrammed into a pluripotent state by retroviral transduction of four transcription factors: OCT4, KLF4, c‐MYC and SOX2.^147^ The reprogrammed cells were termed iPSCs and these are similar to embryonic stem cells in their morphology, proliferation, surface antigens, gene expression and capacity to differentiate into the cell types of the three primordial germ layers. Subsequently, Takahashi et al.^148^ applied the same technology to human adult dermal fibroblasts to generate the first human iPSCs. Following this discovery, other studies have shown that it is possible to generate human iPSCs from other adult somatic cell types, including (but not limited to) peripheral blood^149^ and hair follicles.^150^ Over the past 10 years, an array of protocols has been developed that allow for the differentiation of iPSCs into specific neuronal cell types. These protocols have taken advantage of a wealth of developmental studies that have detailed the steps and molecular cues involved in animal embryology. For example, the first step in the development of the neural tube, neural induction, is assumed to be the default pathway involving the bone morphogenetic proteins (BMPs), Wnt and fibroblast growth factor (FGF) signalling pathways.^151^, ^152^, ^153^ Neural induction leads to a default and primitive anterior identity, which is subsequently patterned by extrinsic morphogens such as Wnts, FGFs, retinoic acid and Sonic Hedgehog, giving rise to forebrain, midbrain, hindbrain or spinal cord domains.

Neuronal differentiation of iPSCs follow the same pathways as in vivo to give rise to mature neuronal populations.^154^ The most efficient neural induction of human iPSCs is achieved by dual inhibition of the SMAD signalling pathway, which involves the synergistic inhibition of the BMP and transforming growth factor‐β pathways to direct pluripotent stem cells down a neuronal linage,^152^ ultimately giving rise to a population of neural progenitors.^152^, ^154^, ^155^ These neural progenitor cells can then be patterned into neuronal cell types with regional identities using specific combinations of morphogens, small molecules, growth factors and transcription factors. It should be noted, however, that protocols for the differentiation of iPSCs into neuronal subtypes are imperfect, often yielding a heterogeneous population of cell types. Nevetheless, depending on the combination and timing of these signals, a variety of neuronal cell types can be obtained, including telecephalic precursors,^156^ midbrain dopaminergic neurons,^157^ basal forebrain cholinergic neurons^158^ and spinal motor neurons,^159^ as well as glial cells, such as astrocytes^160^ oligodendrocytes^161^ and microglia‐like cells.^162^

### 4.2 IPSCs: the advantages and caveats of an in‐vitro model of brain development

Much of the work aiming to identify cellular/molecular mechanisms of steroid action on the brain, cognition and memory has been carried out using animal models. Some studies have manipulated steroid hormones to investigate the effects on human behaviour; however there are very few studies that administered steroid hormones on in vitro human brain tissue or cells. The limitations of animal research have resulted in the slow adoption of steroids as therapeutic agents. Advances in stem cell technologies have resulted in the investigation of steroid hormone interactions in a human system. It is now possible to reprogram human somatic cells into iPSCs to study the closer interaction of steroids with human target cells such as neurons derived from iPSCs. This approach for studying the nervous system has the advantage that these neurons carry the same genetic make‐up of the donor.

The rationale for using iPSC‐derived neuronal tissue as a human system to expedite clinical research is very strong. The human brain develops very differently from the rodent brain and has some human‐specific types of neurons not found in rodents.^163^, ^164^, ^165^ Moreover, some steroid molecules that are effective in humans are not as effective in rodents, whereas some steroid signalling mechanisms are also different in humans than rodents. For example, cortisol is the active glucocorticoid in humans, whereas corticosterone is more active in rodents.^120^ It is also not clear whether the aromatase reaction plays an important role in the developing human brain, and whether testosterone acts via its native receptor AR to masculinise the brain rather than being converted into oestrogens through the aromatase reaction, as observed in animal models. It has also been found that, in humans, aromatase is only expressed in selective brain regions such as the preoptic area, hypothalamus and amygdala.^166^, ^167^ For these reasons, it is essential to develop in vitro human iPSC models to study effects of steroids in humans. It is also possible to accurately characterise the interaction of steroids with human gene products involved in synaptic and dendritic plasticity, and also to identify interactions with genes associated with neuropsychiatric conditions such as autism and schizophrenia. Additionally, investigation of the interactions with disease‐associated mutations will enable an understanding of how the cognition and memory enhancing effects of steroids can be better used as therapeutic agents for neurodegenerative diseases such as Alzheimer's disease and Parkinson's disease, neuropsychiatric conditions such as depression and schizophrenia, and traumatic cerebral and spinal injury.

However, the iPSC method is not without its issues. Reprogramming of highly differentiated somatic cells such as skin cells or hair cells involves the interaction of key transcription factors with epigenetic molecules to transform the chromatin into an open state to promote higher active transcription.^168^, ^169^, ^170^ Most differentiated somatic cells have a closed chromatin state with several highly condensed heterochromatin regions inhibiting the transcription of genes not required to maintain a somatic lineage.^171^ Unfortunately, the reprogramming method is highly inefficient and not completely understood, and iPSCs can sometimes retain lineage‐specific chromatin signatures, thereby creating differentiation biases towards the original somatic cell.^172^ There are also issues with the neuronal differentiation protocols. The monolayer culture method, which is currently the most prevalent method used to generate iPSC‐neurons, employs a dual SMAD inhibition strategy to generate neurons,^152^, ^173^ has several disadvantages. The most important one is it lacks spatial organisation of the brain. Neuronal morphology and gene expression is also heavily dependent on the mechanical properties of cell adhesion to plate surface and neighbouring cells, whereas plating density and cell adhesion substrate determined quantity and types of neurons.^168^

Thankfully, most of the issues are technical and some of them are already being addressed. For example, a 2017 study showed that histone H3K4 methylation patterns associated with somatic lineage identity are a major cause for the inefficiency of transcriptional reprogramming,^174^ suggesting that demethylation of methylated H3K4 in iPSCs might be a possible strategy for increasing reprogramming efficiency. To overcome drawbacks of monolayer cultures, there has been rapid development of methods for generating cerebral organoids, which are able to mimic the three‐dimensional (3D) structure, cell type composition and organisation, and connectivity of the human brain.^175^, ^176^, ^177^ Neurons in organoid cultures are able to demonstrate greater complexity of cellular interaction when undergoing reduced stress compared to monolayer cultures. Naturally occurring cell‐to‐cell contact seen in organoids also helps organise progenitors and neurons in layers typically observed in the human brain.^168^, ^175^, ^176^, ^177^

### 4.3 Studying the actions of steroids in human neurons

To our knowledge, very few studies have directly investigated the actions of steroids in human neurons generated from iPSCs. The cellular mechanisms associated with the clinical phenotypes are also not well understood, especially role of protein kinase‐mediated immediate effects in humans. Moreover, there is little fundamental knowledge of the stoichiometric ratios of steroid biosynthesis pathway components in neuropsychiatric conditions, and very little understanding of why there is an elevated level of steroids associated with these conditions. Human iPSC‐derived neurons provide the tools to carry out controlled experiments aiming to investigate these previously unidentified cellular/molecular mechanisms.

**Figure 2:**
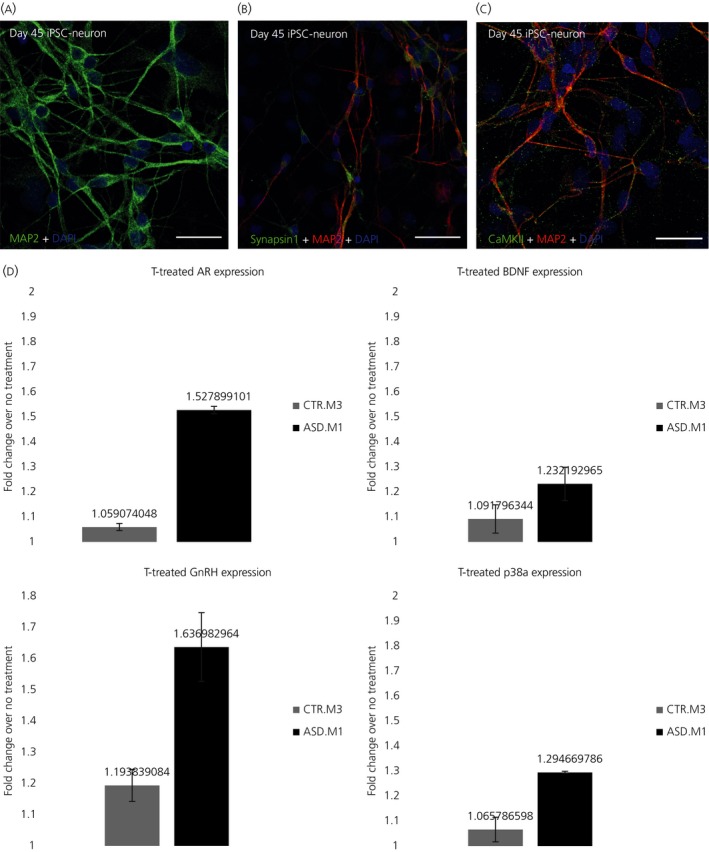
Understanding the effects of testosterone using induced pluripotent stem cell (iPSC)‐neurons derived from typically or atypically‐developing individuals. (A‐C) Example of day 45 human iPSC‐neurons expressing key neuronal and synaptic proteins (scale bar=10 μm). After 45 days, iPSC‐neurons are positive for the microtubule‐associated protein, MAP2 (green) (A), the pre‐synaptic protein, Synapsin‐1 (B) and the post‐synaptic protein Ca^2+^/calmodulin‐dependent protein kinase II (CaMKII) (C). (D) Preliminary data of the effects of testosterone (T) treatment (24 h) on differential gene expression (log‐fold change, *P*≤.05) of androgen receptor (AR), brain‐derived neurotrophic factor (BDNF), gonadotrophin‐releasing hormone (GnRH) and p38a. iPSC lines were generated from typically developed individuals (control: CTR.M3) or atypically developing individuals (ie, patient diagnosed with autism‐spectrum disorder [ASD.M1]). DAPI, 4’,6‐diamidino‐2‐phenylindole. Error bars indicate the SEM

Our group has focused on the generation of fore‐brain‐like, glutamatergic neurons from iPSCs, based on a previously described protocol.^154^ We first generated iPSCs from keratinocytes in hair follicles using a non‐integrating Sendai virus based reprogramming method (CytoTune‐iPS Sendai Reprogramming; Thermo Fisher Scientific Inc., Waltham, MA, USA), then, using the aforementioned neuron differentiation protocol, we generated mature and immature glutamatergic neurons within 40 days; these cells express a range of proteins, including synaptic proteins associated with developing glutamatergic neurons (Figure 2A‐C). Preliminary data demonstrated that male iPSC‐derived immature neurons respond to 17β‐oestradiol exposure. Specifically, 17β‐oestradiol treatment for 24 hours resulted in an increase in neurite outgrowth in these cells.^18^ Interestingly, it was recently reported that 17β‐oestradiol promoted synapse formation between iPSCs‐derived dopaminergic neurons and HEK293 cells and, furthermore, facilitated grafting of these neurons into striatum of a rat model of Parkinson's disease.^178^ Another study by our group, investigating the effect of testosterone on male iPSC‐derived neurons from individuals with autism using a real‐time polymerase chain reaction,^179^ has shown elevated differential expression of AR in autism iPSC‐neurons compared to control iPSC‐derived neurons, after just 24 hours of testosterone administration at physiological levels (2 nmol L^−1^).^180^ Some putative downstream genes were also differentially expressed on testosterone administration, such as brain‐derived neurotrophic factor, gonadotrophin‐releasing hormone and apoptotic marker p38α (Figure 2D). This suggested differential effect of testosterone on autism iPSC‐neurons compared to control neurons after only a short burst of hormone administration. Whether this is indicative of a membrane‐mediated rapid mechanism of action for testosterone remains to be determined in future studies. Further studies will also be necessary to validate these observations, as well as the characterisation of phenotypic changes brought about by testosterone.

### 4.4 Promise of organoids as a tool to study steroid hormone‐brain interactions

**Figure 3:**
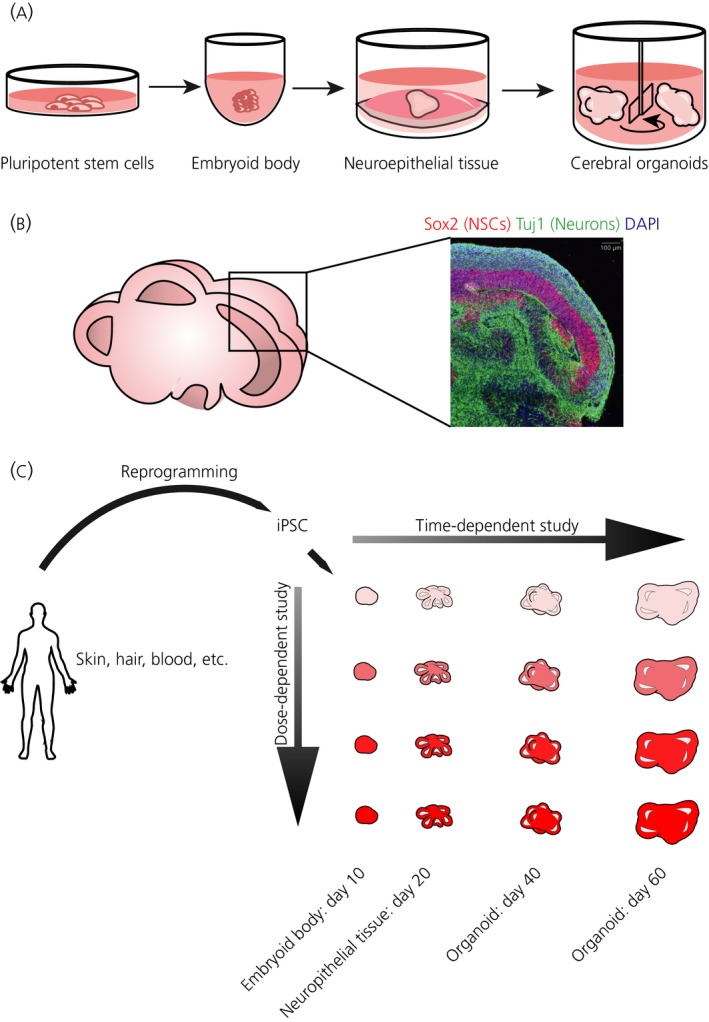
Cerebral organoids recapitulate cortical tissue architecture. (A) Cerebral organoids are generated from pluripotent stem cells, such as induced pluripotent stem cells (iPSCs), which are removed from the two‐dimensional dish and aggregated to form small clumps called embryoid bodies. These begin spontaneously forming embryonic germ layers, and subsequent media selects for neuroepithelial tissue which is placed in a droplet of Matrigel to stimulate outgrowth of neural tube‐like buds. These buds expand and develop into lobes of brain tissue, including the cerebral cortex. (B) Sectioning of cerebral organoids reveals their internal architecture with lobules containing fluid‐filled cavities, much like ventricles, which are lined by neural stem cells (NSCs) (labelled in red by Sox2). Neurons (labelled in green by Tuj1) generated from this ventricular zone then migrate outward and cover the surface of the organoid. DAPI (4’,6‐diamidino‐2‐phenylindole) (blue) labels all cell nuclei. (C) Proposed method to generate “personalised organoids” for steroid hormone research

Cerebral organoids are self‐organised brain tissue derived from stem cells, generated using a patterning growth factor‐free culture method to induce the transformation of embryoid bodies into neuroectoderm. By manipulating growth conditions and environment necessary for mimicking developmental cues, complex brain tissue growth can be established exhibiting heterogeneous regionalisation of different brain areas, including some ventral forebrain regions, dorsal cortex and choroid plexus. Cerebral organoids also form discrete cortical layers with stereotypical inside‐out organisation, as well as human‐specific characteristics such as presence of outer radial glia and matured cortical neurons with various pyramidal identities.^176^ This ability of a 3D cerebral organoid culture to resemble the properties of the developing human brain more closely than monolayer cultures or animal models has made it a preferred model system for undertaking research into the human central nervous system, and it has been proposed to use this method to generate “personalised organoids” to test the effects of steroid hormones and develop drugs^15^, ^181^, ^182^ (Figure 3). Steroid hormones with therapeutic properties such as oestrogens and progesterone can be studied by generating patient‐specific organoids in 3D‐printed mini‐bioreactors, recently developed for the study of the Zika virus on neurodevelopment.^183^ Human brain imaging and animal model studies have shown that testosterone and oestrogens have a well characterised effect on cortical thickness,^184^, ^185^, ^186^ although the regulatory mechanisms are not known and the organoid system is perfectly suited for this kind of study. Sexual differentiation of cortical microstructures can also be studied using high‐throughput organoid cultures.

## 5 CONCLUSIONS

We have reviewed the important role steroids play in development and sexual differentiation of the brain through a nuclear receptor mediated mechanism. Sex‐dependent effects of steroids are long‐term and involve the regulation of transcriptional and epigenetic machinery that brings about permanent changes to brain structures and functions. One well‐characterised mechanism of action is through differential modulation of apoptosis in neurons depending on biological sex. This results in increased size of brain regions such as MPOA and SNB in males, and a larger AVPV in females. The acute, immediate effects of steroids on cognition and memory are evident and act through a cytoplasmic protein kinase‐mediated mechanism. Testosterone and oestrogens are both able to modulate dendritic spine density and structure, at the same time as altering synaptic transmission, although they have different effects on cognition. Testosterone impairs social cognition, and influences autistic traits, whereas oestrogens have neuroprotective effects and enhance memory consolidation and spatial and nonspatial memory tasks. Progesterone, similar to oestrogens, has neuroprotective effects on the brain, although this effect is highly dependent on the timing of exposure. The neuroprotective effect of progesterone against oxidative stress in the hippocampus has a memory enhancing effect, whereas its metabolite, allopregnanolone binds to GABA_A_ receptors increasing inhibition of the CNS and thus disrupting the excitatory‐inhibitory balance. Glucocorticoids, the primary stress hormone, have similar time‐dependent and dose‐dependent effects on the brain. Although high doses of glucocorticoids are harmful to the brain, low levels of glucocorticoid exposure during specific periods of the learning process can significantly enhance memory consolidation, especially if they are related to emotions.

Because steroids and their metabolites have been recurrently proposed as potential therapeutic avenues for a range of neurodevelopmental, neuropsychiatric and neurodegenerative disorders, there is a compelling to supplement and future preclinical studies carried out in animal models, with those carried out in human neurons. However, because many of these disorders have complex aetiologies and underlying molecular and genetic underpinnings, it is not always possible to recapitulate this in animal models. Therefore, translating or refining such findings from preclinical studies into therapies is not straightforward. Deriving iPSCs from specific patient cohorts with similar genetic background and clinical phenotyping, has the potential to improve our understanding of how steroid‐based therapies can be used and, more importantly, possibly improved. Recent developments in iPSC technologies, along with the ability to recapitulate developing 3D brain structures of individual donors, has made it possible to undertake high‐throughput testing of steroid molecules, including several recently discovered neurosteroids that endogenously mediate neuronal functions previously not known to be associated with steroid signalling pathways. Future studies into the synthesis and mechanisms of action of these neurosteroids will further enhance our understanding of the effects of steroids on the brain, and enable us to isolate compounds with greater therapeutic potential for neurodegenerative conditions.
